# Analysis of Psychological Trends and Policy Recommendations of Medical Staff in Northern China in the Latter Stages of the COVID-19 Pandemic

**DOI:** 10.3389/fpsyg.2021.747557

**Published:** 2021-10-20

**Authors:** Xueping Yang, Junxiao Miao, Weizhong Fan, Lili Wang, Shuning Sun, Hongshi Li, Na Wang, Xuesong Wang, Muhui Lin, Ru He

**Affiliations:** ^1^Department of Psychology, The People's Hospital of Liaoning Province, The People's Hospital of China Medical University, Shenyang, China; ^2^Department of Public Places, The Health Supervision Center of Liaoning Province, Shenyang, China; ^3^Department of Cardiology, The People's Hospital of Liaoning Province, The People's Hospital of China Medical University, Shenyang, China; ^4^Department of Neurology, Jinqiu Hospital of Liaoning Province, Shenyang, China; ^5^Department of Oncology and Medical Affair Department, The People's Hospital of Liaoning Province, The People's Hospital of China Medical University, Shenyang, China; ^6^Department of Neurorehabilitation, Second Affiliated Hospital of Zhenzhou University, Zhenzhou, China; ^7^Department of Urinary Surgery, The People's Hospital of Liaoning Province, The People's Hospital of China Medical University, Shenyang, China; ^8^Department of Neurology, The People's Hospital of Liaoning Province, The People's Hospital of China Medical University, Shenyang, China

**Keywords:** psychological status, medical staff, China, COVID-19, anxiety, depression

## Abstract

**Aim:** Since the 2019 coronavirus disease (COVID-19) outbreak, medical staff have faced greater psychological stress and are prone to psychological problems such as anxiety and depression, as confirmed by several studies. This study further clarifies the psychological status of Chinese medical staff during the stable phase of the pandemic through a cross-sectional investigation in a large population sample in northern China.

**Methods:** Subjects: Clinical frontline medical staff from seven hospitals in Liaoning Province were recruited from November 2020 to February 2021.

**Research Tools:** The research tools used were the Self-Rating Anxiety Scale (SAS), Self-Rating Depression Scale (SDS), Simplified Coping Style Questionnaire (SCSQ), and General Status Questionnaire.

**Statistical Analysis:** SPSS 22.0, ANOVA variance analysis, and multiple logistics regression were used for statistical analysis. *P*-values of <0.05 indicated significant statistical differences.

**Results:** A total of 3,144 medical staff completed the survey (599 men [19.1%] and 2,545 women [80.9%]; 1,020 doctors [32.4%] and 2,124 nurses [67.6%]). Among all subjects, the rates of anxiety and depression were 21.1% (663/3, 144) and 43.9% (1,381/3,144), respectively. Multiple logistic comparative analysis revealed that age (OR = 1.272, 95% CI = 1.036–1.561, *P* = 0.022), the need for psychological counseling (OR = 1.566, 95% CI = 1.339–1.830, *P* < 0.001), and the coexistence of depression (OR = 0.050, 95% CI = 0.038–0.066, *P* < 0.001) were significantly associated with anxiety. Coexisting anxiety was also associated with the occurrence of depression (OR = 0.050, 95% CI = 0.038–0.065, *P* < 0.001).

**Conclusions:** In the later stages of the pandemic in China, the occurrence rates of anxiety and depression among medical staff remain high. In addition to age, there is little correlation between anxiety or depression and general factors such as gender and profession. As a special group, medical staff show different psychological changes at various times during a stressful event. Concerning for the psychological needs of medical staff and different psychologically oriented policy implementation are needed.

## Introduction

Since the end of December 2019, a severe acute respiratory disease known as the novel coronavirus spread across several countries, causing a major global outbreak. The World Health Organization (WHO) officially named the disease the 2019 novel coronavirus disease, or COVID-19, on February 11, 2020. COVID-19 is a potential zoonotic disease with low to moderate mortality rate (about 2–5%). Human-to-human transmission occurs through droplet inhalation or contact transmission through droplets, which endangers frontline medical staff (Wu, Y. C., et al., 2020). COVID-19 has a serious impact not only on physical health but also on mental health (i.e., increased anxiety, depressive symptoms, and insomnia), which also affects daily behaviors, economic and preventive decision-making by government officials, and the operation of medical institutions and centers (Torales et al., 2020).

In China, at the beginning of the pandemic, medical staff on the frontline actively displayed professional skills and played an important role in the prevention and control of the pandemic. However, the occurrence of COVID-19 was sudden and uncertain and coupled with work and environmental pressures. Thus, medical staff, especially frontline workers, often faced increased psychological stress and experienced burnout characterized by emotional exhaustion and/or depersonalization even Burnout syndrome (BO), (Baro Vila et al., 2021; Cyr et al., 2021), such as anxiety about infection, leading to the occurrence of psychological problems in many countries including the countries with special backgrounds such as war (Elhadi et al., 2020b). A meta-analysis of several studies from multiple countries, including China, suggested that the rates of anxiety and depression remain the most severe psychological problem among populations such as medical staff, the general population, and COVID-19 patients (Luo et al., 2020).

Since the beginning of the COVID-19 pandemic, studies of medical staff have been conducted in China, and it has been found that the occurrence of psychological problems is high. Specifically, rates are higher in medical professionals than in non-clinical workers, the general population, and college students (Huang et al., 2020; Wu, W., et al., 2020; Zhang et al., 2020; Zhu et al., 2020). Frontline medical staff are more likely to feel fear, depression, and anxiety, particularly those who are exposed to infection and working in departments such as respiratory, emergency, infectious disease, and ICUs (Lu et al., 2020).

The mental health of medical staff should be emphasized, and further psychological measures and targeted research are needed. From the beginning of the crisis to the current normalized management of the pandemic, the psychological changes of medical staff also show some trends and characteristics. On the basis of previous research, in this study, the psychological status changes in medical staff in Liaoning Province in the latter stages of the pandemic are analyzed and interpreted. The impact of related factors on depression and anxiety is further clarified, and the relationship between psychological change and psychological response is discussed. These methods are achieved by studying the combination of specific data, adjustments in government policy, and specific policy changes in hospitals. This paper provides scientific advice for subsequent preventive and coping measures and scientific support for further related research.

## Methods

### Subjects

From November 2020 to February 2021, clinical frontline medical staff working in seven general and psychiatric specialties hospitals (four general hospitals and three psychiatric specialties hospitals) were from five cities of Liaoning Province including the provincial capital city (Shenyang). They were investigated randomly in a cross-sectional study. The clinical frontline medical staff defined in this study is engaged in the clinical work, and involved in the examination and treatment of patients directly or indirectly. All the participants in this study are all clinical frontline medical staff. It was needed to meet the inclusion criteria: 18 years of age or older, from clinical departments, having good educational level and comprehension, and the exclusion criteria: no serious physical or mental illness, and no mental developmental disorders. All the participants provided their written informed consent and all information about the participants was confidential. The study was approved by the Ethics Committee of Liaoning Provincial People's Hospital.

### Research Tools

Online psychological scale surveys “Questionnaire Star” served as the research tools, which included the following scales: Self-Rating Anxiety (SAS; standard score of ≥50 points indicates symptoms of anxiety, and higher scores indicate more severe anxiety); Self-Rating Depression Scale (SDS; standard score of ≥50 points indicates depressive symptoms, higher scores indicate more severe depression); and Simplified Coping Style Questionnaire (SCSQ; a total of 20 entries, score of 0–60, after conversion, response tendency value of >0 indicates that the subject under stress mainly had a positive response to the stress, value of <0 indicates adoption of negative coping style). A general status questionnaire and investigation of the situation related to COVID-19 were used in the study.

### Statistical Analysis

Statistical analysis was performed using SPSS 22.0 statistical package (SPSS Inc., Chicago). The sociodemographic characteristics of participants and outbreak-related issues were descripted by frequency analysis. The SAS, SDS, and positive and negative coping scores (M ± SD) were compared across different groups. All variances analysis in anxiety and depression groups of the whole sample was followed by a single-factor analysis (ANOVA). Moreover, multiple logistics regression analysis was used to assess the association between outcome variables (the reported level of anxiety and depression) and potential factors affecting the occurrence of anxiety and depression. The results were indicated by frequency, percentage (%), and average and standard difference (mean ± SD). *P*-values of <0.05 indicated significant statistical differences.

## Results

A total of 3,144 out of 3,295 medical staff (95.42%) successfully completed the survey (age range: 19–66 years, average age: 36.50 ± 11.03 years), including 599 men (19.1%), 2,545 women (80.9%), 1,020 doctors (32.4%), and 2,124 nurses (67.6%). The rate of no response was 4.58% and there was no effect on the final statistics. The subjects covered internal medicine (803, 25.5%), surgery (354, 11.3%), department of ophthalmology and otorhinolaryngology (47, 1.5%), obstetrics and gynecology (102, 3.2%), pediatrics (49, 1.6%), psychiatric (1,154, 36.7%) and other professional departments (635, 20.2%). The general characteristics of subjects were as follows, as shown in Table 1.

**Table 1:** Sociodemographic characteristics of participants and outbreak-related issues (*N* = 3,144).

**Variables**	**No. (%)**
**Gender**	
Males	599 (19.1%)
Females	2,545 (80.9%)
**Age**	
18–28	697 (22.2)
29–44	1,663 (52.9%)
45–60	780 (24.8)
60 years old above	4 (0.1%)
**Smoking history**	
No	2,932 (93.3%)
Less or 10/day	113 (3.6%)
More than 10 /day	99 (3.1%)
**Alcohol drinking history**	
No	2,730 (86.8%)
Less or 3 times/week	374 (11.9%)
More than 3 times/week	40 (1.3%)
**Status of only child**	
Yes	1,623 (51.6%)
No	1,521 (48.4%)
**Marital status**	
Unmarried	759 (24.1%)
Married	2,265 (72.0%)
Divorced	110 (3.5%)
Widowed	10 (0.3%)
**Educational level**	
Below undergraduates	603 (19.2%)
Undergraduates	2,093 (66.6%)
Above undergraduates	448 (14.2%)
**The number of parenting children**	
None	1,150 (36.6%)
One child	1,783 (56.7%)
Two and above	211 (6.7%)
**Family history of mental illness**	
Yes	69 (2.2%)
No	3,075 (97.8%)
**Work position**	
Doctors	1,020 (32.4%)
Nurses	2,124 (67.6%)
**Department-professions**	
Internal medicine	803 (25.5%)
Surgery	354 (11.3%)
Ophthalmology and otorhinolaryngology	47 (1.5%)
Obstetrics and gynecology	102 (3.2%)
Pediatrics	49 (1.6%)
Psychiatric	1,154 (36.7%)
Others	635 (20.2%)
**The change and affecting factors about COVID-19**	
Work position change during the outbreak	
Stick to previous department	2,712 (84.2%)
Full-time in fever outpatient department	100 (3.1%)
Participated in fever outpatient department	91 (2.8%)
Others	176 (5.5%)
Assisted Hubei's work	65 (2.0%)
**Impact on own work after COVID-19 outbreak**	
No	358 (11.4%)
Slight	1,171 (37.2%)
Significant	1,313 (41.8%)
Huge	302 (9.6%)
**Fear of infection**	
Yes	1,585 (50.4%)
No	1,559 (49.6%)
**The need for psychological counseling**	
No need	958 (30.5%)
Sufficient	1,857 (59.1%)
Not sufficient	329 (10.5%)

Among the subjects meeting the anxiety and depression score, SAS, SDS, and positive and negative coping scores were compared across different groups (Table 2), followed by a single-factor ANOVA analysis of the whole sample (Table 3). In the ANOVA analysis of anxiety, the number of parenting children (*F* = 3.457, *P* = 0.032), need for psychological counseling (*F* = 139.793, *P* < 0.001), coexistence of depression (*F* = 1,813.251, *P* < 0.001), and positive or negative coping style (*F* = 3.918, *P* = 0.048) were significantly related to anxiety scores. In the ANOVA analysis of depression, only the need for psychological counseling (*F* = 52.962, *P* < 0.001) and coexistence of anxiety (*F* = 1,213.181, *P* < 0.001) were significantly related to depression scores.

**Table 2:** Comparison of scores between the anxiety and depression subgroups, health subgroups, and whole sample.

**Mean ± standard difference**	**The whole sample**	**Anxiety**		**Depression**	
**M ± SD**	***N* = 3,144**	**Anxiety subjects**	**No anxiety subjects**	**Depression subjects**	**No depression subjects**
		**663 (21.1%)**	**2,481(78.9%)**	**1,381 (43.9%)**	**1,763 (56.1%)**
SAS	43.32 ± 10.26	58.69 ± 7.81	37.21 ± 6.08	50.33 ± 9.59	37.83 ± 6.85
SDS	47.33 ± 13.40	61.01 ± 8.69	43.68 ± 12.00	60.46 ± 6.25	37.04 ± 6.96
Positive coping average scores	1.80 ± 0.61	1.54 ± 0.59	1.87 ± 0.60	1.61 ± 0.63	1.96 ± 0.54
Negative coping average scores	1.11 ± 0.56	1.30 ± 0.56	1.06 ± 0.54	1.19 ± 0.61	1.06 ± 0.51

**Table 3:** Single-factor analysis of potential factors and anxiety/depression scale scores.

**Variables**	**The anxiety scores of whole sample**			**The depression scores of whole sample**		
	**(X±s)**	**T/F**	***P***	**(X±s)**	**T/F**	***P***
**Gender**						
Males	43.25 ± 10.54			47.32 ± 13.14		
Females	43.33 ± 10.19	0.030	0.862	47.33 ± 13.46	0.001	0.977
**Age**						
18–28	42.54 ± 10.15			46.60 ± 13.32		
29–44	43.53 ± 10.46			47.56 ± 13.45		
45–60	43.58 ± 9.89			47.54 ± 13.33		
60 years old above	39.68 ± 5.72	1.903	0.127	36.63 ± 10.08	1.942	0.121
**Smoking history**						
No	43.32 ± 10.20			47.30 ± 13.43		
Less or 10/day	43.26 ± 11.18			47.93 ± 12.97		
More than 10 /day	43.24 ± 10.98	0.005	0.995	47.44 ± 12.98	0.123	0.885
**Alcohol drinking history**						
No	43.39 ± 10.27			47.39 ± 13.47		
Less or 3 times/week	43.05 ± 10.06			47.22 ± 12.69		
More than 3 times/week	41.25 ± 10.99	0.999	0.368	44.63 ± 15.03	0.853	0.426
**Status of only child**						
Yes	43.40 ± 10.25			47.68 ± 13.39		
No	43.23 ± 10.26	0.231	0.631	46.96 ± 13.40	2.23	0.135
**Marital status**						
Unmarried	42.88 ± 10.22			46.55 ± 13.17		
Married	43.44 ± 10.29			47.51 ± 13.47		
Divorced	44.16 ± 9.93			49.26 ± 13.06		
Widowed	40.38 ± 8.94	1.085	0.354	45.75 ± 15.93	1.797	0.145
**Educational lever**						
Below undergraduates	42.99 ± 9.66			46.96 ± 13.11		
Undergraduates	43.46 ± 10.40			47.46 ± 13.49		
Above undergraduates	43.09 ± 10.38	0.642	0.526	47.21 ± 13.38	0.351	0.704
**The number of parenting children**						
None	42.96 ± 10.10			46.93 ± 13.31		
One child	43.70 ± 10.41			47.68 ± 13.43		
Two and above	42.08 ± 9.65	3.457	**0.032**	46.54 ± 13.54	1.495	0.224
**History of mental illness**						
Yes	43.77 ± 9.46			49.02 ± 12.88		
No	43.31 ± 10.27	0.135	0.713	47.29 ± 13.41	1.124	0.289
**Work position**						
Doctors	43.29 ± 10.16			47.60 ± 13.45		
Nurses	43.33 ± 10.31	0.012	0.914	47.20 ± 13.37	0.613	0.434
**Department-professions**						
Internal medicine	43.11 ± 10.30			47.23 ± 13.64		
Surgery	43.17 ± 9.75			46.96 ± 13.26		
Ophthalmology and otorhinolaryngology	47.37 ± 12.48			50.43 ± 12.40		
Obstetrics and gynecology	42.72 ± 8.79			47.92 ± 13.29		
Pediatrics	41.68 ± 8.96			43.49 ± 13.57		
Psychiatric	43.34 ± 10.59			47.08 ± 13.45		
Others	43.56 ± 9.95	1.614	0.139	48.09 ± 13.09	1.588	0.147
**The change and affecting factors about COVID-19**						
Work position change during the outbreak						
Stick to previous department	43.36 ± 10.15			47.44 ± 13.34		
Full-time in fever outpatient	43.53 ± 11.03			47.86 ± 13.40		
Participated in fever outpatient	42.76 ± 11.17			46.52 ± 14.48		
Others	43.27 ± 11.27			46.38 ± 13.54		
Assisted Hubei's work	42.38 ± 9.52	0.222	0.927	45.46 ± 14.08	0.706	0.588
**Impact on own work after COVID-19 outbreak**						
No	43.02 ± 10.79			47.31 ± 14.08		
Slight	42.97 ± 9.71			46.97 ± 13.35		
Significant	43.63 ± 10.49			47.30 ± 13.25		
Huge	43.64 ± 10.62	1.051	0.369	48.86 ± 13.37	1.589	0.190
**Fear of infection**						
Yes	43.24 ± 9.91			47.46 ± 13.03		
No	43.39 ± 10.60	0.169	0.681	47.20 ± 13.77	0.297	0.586
**The need for psychological counseling**						
No need	43.64 ± 10.29			47.51 ± 13.06		
Sufficient	41.70 ± 9.02			46.03 ± 13.20		
Not sufficient	51.51 ± 12.48	139.793	**<0.001**	54.14 ± 13.39	52.962	**<0.001**
**Depression/anxiety coexisting or no**						
Yes	50.33 ± 9.59			61.01 ± 8.69		
No	37.83 ± 6.85	1,813.251	**<0.001**	43.48 ± 12.00	1,213.181	**<0.001**
**Coping style**						
Positive	42.95 ± 9.96			47.06 ± 13.26		
Negative	43.67 ± 10.52	3.918	**0.048**	47.59 ± 13.53	1.206	0.272

*The values shown in bold in the table indicate that the p-value is less than 0.05*.

Among all subjects, the rate of anxiety was 21.1% (663/3,144; average score: 58.69 ± 7.81), of which 217 were doctors (32.7%), and 446 (67.3%) were nurses. The rate of depression was 43.9% (1,381/3,144; average score: 60.47 ± 6.24), of which 350 (25.3%) were doctors and 1,031 (74.7%) were nurses. In the sample of doctors, the rates of anxiety and depression were 21.3% (217/1,020) and 44.8% (457/1,020), respectively. In the sample of nurses, the rates of anxiety and depression were 21.0% (446/2,124) and 43.5% (924/2,124), respectively.

In further multiple logistic analysis (Table 4), age factor (*R* = 1.272, 95% CI = 1.036–1.561, *P* = 0.022), the need for psychological counseling (OR = 1.566, 95% CI = 1.339–1.830, *P* < 0.001), and the coexistence of depression (OR = 0.050, 95% CI = 0.038–0.066, *P* < 0.001) were significantly associated with anxiety. Coexisting anxiety was also associated with the occurrence of depression (OR = 0.050, 95% CI = 0.038–0.065, *P* < 0.001).

**Table 4:** Multiple logistics analysis of independently related factors of anxiety and depression.

**Variables**	**Anxiety**				**Depression**			
	**Anxiety subjects *N*(%)**	**OR**	**95% Cl**	***P*-value**	**Depression subjects *N*(%)**	**OR**	**95% Cl**	***P*-value**
**Gender**								
Males	124 (20.7%)				258 (43.1%)			
Females	539 (21.2%)	0.918	0.691–1.219	0.554	1,123 (44.1%)	1.075	0.857–1.349	0.531
**Age**								
18–28	113 (16.2%)				290(41.6%)			
29–44	383 (23.0%)				753(45.3%)			
45–60	167 (21.4%)				338(43.3%)			
60 years old above	0 (0%)	1.272	1.036–1.561	**0.022**	0(0%)	0.928	0.789–1.090	0.361
**Smoking history**								
No	618 (21.1%)				1,286(43.9%)			
Less or 10/day	23 (20.4%)				52(46.0%)			
More than 10 /day	22 (22.2%)	1.136	0.771–1.674	0.520	43(43.4%)	0.915	0.668–1.253	0.579
**Alcohol drinking history**								
No	583 (21.4%)				1,209 (44.2%)			
Less or 3 times/week	74 (19.8%)				160 (42.8%			
More than 3 times/week	6 (15.0%)	1.089	0.796–1.490	0.594	12 (30.0%)	0.951	0.746–1.213	0.688
**Status of only child**								
Yes	336 (20.7%)				731 (45.04%)			
No	327 (21.5%)	1.061	0.852–1.321	0.597	650 (42.7%)	0.870	0.729–1.039	0.124
**Marital status**								
Unmarried	143 (18.8%)				314 (41.4%)			
Married	490 (21.6%)				1,009 (44.5%)			
Divorced	28 (25.5%)				54 (49.1%)			
Widowed	2 (50.0%)	0.946	0.726–1.234	0.684	4 (40.0%)	1.179	0.951–1.463	0.133
**Educational lever**								
Below undergraduates	111 (18.4%)				248 (41.1%)			
Undergraduates	457 (21.8%)				934 (44.6%)			
Above undergraduates	95(21.2%)	1.222	0.977–1.528	0.079	199 (44.4%)	0.997	0.836–1.188	0.971
**The number of parenting children**								
None	215 (18.7%)				493 (42.9%)			
One child	409 (22.9%)				797 (44.7%)			
Two and above	39 (18.5%)	1.048	0.846–1.298	0.671	91 (43.1%)	0.940	0.790–1.118	0.482
**Family history of mental illness**								
Yes	15 (21.7%)				35 (50.7%)			
No	648 (21.1)%	1.133	0.589–2.180	0.709	1,346 (43.8%)	0.714	0.418–1.222	0.220
**Work position**								
Doctors	217 (21.3%)				457(44.8%)			
Nurses	446 (21.0%)	1.277	0.967–1.686	0.085	924(43.5%)	0.897	0.718–1.121	0.340
**Department-professions**								
Internal medicine	167 (20.8%)				358(44.6%)			
Surgery	80 (22.6%)				146(41.2%)			
Ophthalmology and otorhinolaryngology	15 (31.9%)				25(53.2%)			
Obstetrics and gynecology	16 (15.7%)				47(46.1%)			
Pediatrics	7 (14.3%)				16(32.7%)			
Psychiatric	250 (21.7%)				491(42.5%)			
Others	128 (20.2%)	0.994	0.954–1.037	0.785	298(46.9%)	1.009	0.975–1.045	0.591
**The change and affecting factors about COVID-19**								
Work position change during the outbreak								
Stick to previous department	570 (21.01%)				1,195(44.1%)			
Full-time in fever outpatient	25 (25.0%)				45(11.3%)			
Participated in fever outpatient	19(20.9%)				41(45.1%)			
Others	39(22.2%)				74(42.0%)			
Assisted Hubei's work	10(15.4%)	1.010	0.926–1.101	0.830	26(40.0%)	0.976	0.910–1.046	0.489
**Impact on own work after COVID-19 outbreak**								
No	72(20.1%)				158(44.1%)			
Slight	231 (19.7%)				504(43.0%)			
Significant	297 (22.6%)				568(43.3%)			
Huge	63 (20.9%)	1.050	0.925–1.193	0.448	151(50.0%)	1.023	0.922–1.136	0.670
**Fear of infection**								
Yes	333 (21.0%)				707(44.6%)			
No	330 (21.2%)	1.103	0.893–1.364	0.363	674(43.2%)	0.928	0.781–1.102	0.393
**The need for psychological counseling**								
No need	210 (21.9%)				416(43.4%)			
Sufficient	276 (14.9%)				755(40.7%)			
Not sufficient	177 (53.8%)	1.566	1.339–1.830	**<0.001**	210(63.8%)	1.061	0.922–1.221	0.407
**Depression/anxiety coexisting or no**								
Yes 1381 663	598 (43.3%)				598(90.2%)			
No 1763 2481	65 (3.7%)	0.050	0.038–0.066	**<0.001**	783(31.6%)	0.050	0.038–0.065	**<0.001**
**Coping style**								
Positive 1543	310 (20.1%)				676(43.8%)			
Negative 1601	353 (22.0%)	1.185	0.971–1.446	0.094	705(44.0%)	0.952	0.810–1.118	0.548

*The values shown in bold in the table indicate that the p-value is less than 0.05*.

Although the rate of anxiety in males was lower than that in females (124/599, 20.7% vs. 539/2,545, 21.2%), the rate of depression in males was lower than in females (258/599, 43.1% vs. 1,123/2,545, 44.1%). However, statistical analysis showed no statistical differences between gender and the occurrence of anxiety and depression (*P*-values were 0.554 and 0.531, respectively). Regarding profession, the highest rates of anxiety and depression occurred in the ophthalmology and otorhinolaryngology departments at 31.9% (15/47) and 53.2% (25/47), respectively. The pediatric department showed the lowest rates of anxiety and depression at 14.3% (7/49) and 32.7% (16/49), respectively.

The sample sizes of the departments with the highest and lowest rates of anxiety and depression were both small. Statistical analysis revealed no statistical differences between the different departmental specialty groups and levels of anxiety (*R* = 0.994, 95% CI = 0.954–1.037, *P* = 0.785) or depression (*R* = 1.009, 95% CI = 0.975–1.045, *P* = 0.591). Family history of mental illness was not related to anxiety or depression (*P*-values were 0.709 and 0.220, respectively). There were no significant statistical differences between coping style and the anxiety-free (*P* = 0.094) or depression-free (*P* = 0.548) group, and the other factors were not related to the occurrence of anxiety or depression (Table 4).

## Discussion

In China, mental health problems caused by COVID-19 have brought great challenges to traditional forms of mental health services, but this has also brought new development and change. However, based on past prevention measures and management of mental health and mental illness in China, these experiences may be beneficial to the prevention and control of COVID-19 and to other countries. Relevant studies can provide a scientific basis for further research and policy formulation. For medical staff, especially psychiatrists, who were not only impacted by COVID-19 but also served as implementers of specific policies, mental status should be emphasized. Through this investigative research, the psychological status of medical staff in Liaoning Province was elucidated, and some useful data were provided. The following analysis was performed by combining past comparative studies with the status quo of prevention and control measures.

### Comparative Analysis of Past Studies and Related Factors

Previous studies have shown that the rates of depression and anxiety in medical staff were higher than other populations, although they were lower than those in COVID-19 patients. For example, the prevalence of depression was 43.1% in COVID-19 patients in Hubei Province Isolation Hospital (Ma et al., 2020). In a small sample of patients, the risk of anxiety and depression was even higher (55.3% and 60.2%, respectively), and depressive symptoms were more prominent than anxiety (Guo, Q., et al., 2020). Compared to other populations, some studies performed in the early stages of the 2020 COVID-19 outbreak showed that the prevalence of anxiety and depression in adults in isolated populations from large families and communities was 32.7% (Guo, Y., et al., 2020).

Additionally, the prevalence of post-traumatic stress disorder (PTSD) and depression among college students was 2.7% and 9.0%, respectively. But due to special background effects such as war, the incidence of anxiety and depression among the college students during the COVID-19 outbreak may be very high (64.5 and 88%, respectively), (Elhadi et al., 2020a). The rates of anxiety and depression were 14 and 19% in the general public in Hong Kong, respectively (Choi et al., 2020; Tang et al., 2020). For medical staff, a certain percentage of medical staff had anxiety, depression, burnout or clinically-relevant psychosocial distress, confirmed by investigation studies conducted in other countries such Kenya, Japan, and Canada (Binnie et al., 2021; Kwobah et al., 2021; Matsuo et al., 2021). In this study, the rates of anxiety and depression were 21.1 and 43.9% in the whole sample, respectively, which were lower than those in COVID-19 patients or the people with a special background and higher than those in other populations. It was found that the factors associated with COVID-19 infection are still important to the occurrence of anxiety and depression. The findings regarding the medical staff are also similar to the results of a previous study by Huang et al. The incidences of anxiety and PTSD were 23.04 and 27.39%, respectively (Huang et al., 2020). This study also found that depressive symptoms were more pronounced than anxiety symptoms and the coexistence of depression was significantly associated with the occurrence of anxiety (*P* < 0.001). It revealed that depression and anxiety were reciprocal risk factors and that the presence of anxiety and depression increased the risk of reoccurrence or coexistence of both depression and anxiety.

Additionally, it was found that work stress of Chinese medical staff during the past severe acute respiratory syndrome (SARS) period was linked to concerns about infection and corresponded to findings in studies with larger samples. Increased work stress and fear of infection are still causes of worsened mental status (Tian et al., 2003). A February 2020 study by Liu et al. that surveyed medical staff in Hubei Province found that direct contact between COVID-19 patients was an important factor in the occurrence of anxiety, but socio-demographic characteristics (i.e., gender, age, education, and marital status) were not (Liu et al., 2020), which suggests that the medical staff's concerns were related to infection. In this study, about half (50.4%) of medical staff were concerned about infection, but statistical analysis showed that this was not significantly associated with anxiety or depression (*P*-values were 0.363 and 0.393, respectively). These results may be related to improved progress and adjustments in prevention and control measures, as well as the rapid improvements in COVID-19 protective measures, which both reduced the risk and fear of infection among medical staff.

Previous studies have found that other factors are associated with anxiety and depressive symptoms, such as age, reduced income, other diseases, COVID-19 infection, living alone, family conflicts, sleep problems. A study by Guo Yan et al. also showed that people at high risk of anxiety or depressive symptoms or COVID-19 patients needed urgent psychological support during the COVID-19 pandemic, including online psychological support, relaxation training, and online consulting (Guo, Q., et al., 2020; Guo, Y., et al., 2020). In this study, part of the medical staff showed a need for psychological counseling. A total of 329 medical staff (10.5%) were not eligible for existing counseling, which was significantly correlated with the occurrence of anxiety (*P* < 0.001) but not with the occurrence of depression (*P* = 0.407). This may be because the need for psychological counseling of the more anxious medical staff was stronger. Previous studies have also found that the occurrence of adverse psychological problems coexists with obvious gender and work position characteristics. For example, the occurrence rate of adverse psychological problems in nurses was higher than doctors, and the occurrence of adverse psychological problems in women was higher than men (Huang et al., 2020; Binnie et al., 2021; Kwobah et al., 2021). However, in this study, the rates of anxiety (20.7 vs. 21.2%) and depression (43.1 vs. 44.1%) in men and women were similar, and the rates of anxiety (21.3 vs. 21.0%) and depression (44.8 vs. 43.5%) in doctors and nurses were also similar.

In further statistical analysis, gender and work position were not significantly correlated with anxiety (*P*-values were 0.554 and 0.085, respectively) or depression (*P*-values were 0.531 and 0.340, respectively). This indicates that gender and work position are not the main factors affecting the occurrence of anxiety or depression. In this study, age was the only general factor related to the occurrence of anxiety (*P* = 0.022), and other factors (i.e., status of only child, educational level) were not related to the occurrence of anxiety or depression. However, the results also provide insight into interventions that may better mitigate risk factors and warrant further study of other relevant factors.

As for the impact of profession, previous longitudinal studies have found that after the outbreak of COVID-19, some professions, such as surgeons, experienced higher prevalence of anxiety and depression compared to the past (Xu et al., 2020). In this study, because of the lack of basic research data before the outbreak, it is not possible to draw accurate and scientific conclusions. However, in horizontal comparisons between different professions, previous studies have shown that clinicians display higher levels of anxiety and depression than non-clinical staff, especially those in departments working closely with infected patients (Lu et al., 2020). However, there was no detailed and specific analysis of the relationship between specific profession and the occurrence of psychological problems.

In this study, the rates of anxiety and depression were highest in the ophthalmology and otorhinolaryngology department (31.9 and 53.2%, respectively) and lowest in the pediatric department (14.3 and 32.7%, respectively). However, statistical analysis found no correlation between professional specialty and anxiety or depression (*P*-values were 0.785 and 0.591, respectively). Work position change during the outbreak was also not related to anxiety or depression (*P*-values were 0.830 and 0.489, respectively), suggesting that profession and change in work position were not main factors in the later period of the COVID-19. However, the bias may have been caused because the sample sizes of the professional department were small and because the sample distribution was not scientific.

Research data from studies of medical staff in Germany, Poland, France, and other countries found that depression and anxiety were associated with factors such as age, gender, occupation, profession, type of activity, intensity of work stress, proximity to COVID-19 patients (Azoulay et al., 2020; Bohlken et al., 2020; Hoseinabadi et al., 2020; Maciaszek et al., 2020). In the study described here, it was found that the above factors, including age, are not related to the occurrence of anxiety or depression. This may be due to specific national conditions, race, genetics, personality characteristics, timing of the research, and different sample sizes. However, impacts of the intensity and pressure of work are also consistent with the later analysis of policy impact; the decline of depression, anxiety, and other psychological problems that declined in the later stages of the pandemic was due to the reduction in stress-related workload and work intensity. However, the relevant data from this study can serve as a reference for further research, more comprehensive assessment of risk factors, and scientific policy formulation.

### Incidence of Psychological Conditions From the Perspective of Stress

Differences in individual psychological stress patterns may lead to different individual responses to COVID-19 outbreak stress. Stress refers to the response to harmful stimuli by an organism. Psychological stress is a process of adaptation or response in an individual when perceiving (through cognition and evaluation) a threat or challenge from environmental change (stress source), and it is a multifactorial, interactive system. With changes in modern medical models of biology-psychology-society, it has been found that the mechanisms of disease can involve important psychological factors, which broadens the vision of disease prevention and treatment (Tang, 2012). Therefore, the occurrence of many diseases is related to psychological stress patterns. By combining methods used in studying current pandemic prevention and control and related research, changes in individual psychological status have been found to be related to psychological stress patterns. However, in this study, individual coping style was not related to the occurrence of anxiety or depression (*P*-values were 0.094 and 0.548, respectively). Additionally, only a relationship between coping style and anxiety was found in a single-factor analysis (*P* = 0.048).

No supporting evidence was found regarding the effects of individual stress response pattern on the occurrence of depression and anxiety. However, the improvements in individual psychological quality level and stress pattern maybe reduce the occurrence of adverse psychological problems. It was also found in this study that educational level was not related to the occurrence of anxiety or depression (*P*-values were 0.079 and 0.971, respectively). This suggests that general education cannot replace mental health education and training, which is still necessary. For example, the popularization of continuing education may also improve the psychological ability to combat stress experienced by medical staff, regarding the knowledge and skills related to psychology, mental disorders, and psychological crisis intervention. In the future, in-depth research about more personality characteristics should be conducted, which can serve as a reference for other countries.

### Psychological Impact of Government Policy Measures and Hospital Prevention and Control Pattern on Medical Staff

After the outbreak, hospitals in China set up an emergency fever outpatient department, in which patients with fever were specially managed. Emergency channels were opened normally, but patients with general disease status were recommended a choice of treatment. Some medical staff assisted Hubei's work in the wards with COVID-19 patients and outpatient individuals with fever. Medical staff faced a higher risk of infection and showed a higher risk of depression and anxiety than non-clinical staff. This was due to special working conditions, work intensity, and separation from families, especially for the frontline medical staff fighting COVID-19 (Wu and Wei, 2020).

Since the outbreak began, policies were quickly adjusted, and medical staff learned about constantly changing COVID-19-related guidelines through continuing education and hospital norms, carding and perfecting diagnoses, and adhering to treatment and protection standards. The policies helped strengthen the understanding of COVID-19. Besides, the measures played an important role in reducing the risk of anxiety and depression, strengthened the use of proper protective equipment, reduced infection risk, encouraged more reasonable scheduling, substantially reduced the number of confirmed and suspected patients, reduced work intensity, and promoted good labor compensation and life assistance. Some factors affecting the psychological status of medical staff have also been studied by Zhou et al. (Zhou et al., 2020). Moreover, teamwork was used in both routine prevention and control work or assistance to hospitals in Hubei. Working together provided a good atmosphere for medical staff and also satisfied the psychological need for human support. All of the above techniques are important measures for the improvement of mood in medical staff.

During more stable stages, the deployment of COVID-19 prevention and control measure was more scientific, and social productivity began to resume, with the improvement of public environment. While emphasizing the more normalized measures fighting COVID-19 in hospitals, more medical staff covered routine work, and specialized professionals rotated duty regarding COVID-19 prevention and control work. However, this study showed that the psychological status of medical staff should be a focus during the stable phase of the pandemic. In this study, the occurrence of anxiety and depression was weakly associated with general socio-demographic characteristics. Therefore, from the aspect of policy control, changes in psychological status were consistent with the improvements and implementation in prevention and control system as well as policy.

Regarding the occurrence of major health events, the government's decision-making as well as the improvement of counseling systems for meeting psychological needs play an important role in adjusting social order, reducing the stress levels of the public and medical staff, and reducing the occurrence of psychological problems. At the same time, psychological screening systems in hospitals should be improved, and routine psychological screening after major public health events should be conducted. Staff members struggling with their psychological well-being should be further identified and given special psychological intervention, if necessary.

### Psychological Intervention

At all points during an outbreak, attention to individual's psychological needs is necessary. In the early stages of the pandemic, a psychological consulting hotline was set up to provide psychological help to the general public, as well as online and offline guidance and consultation for medical personnel with depression and anxiety. In Hubei, medical staff were specially equipped with psychologists, individual and group interventions, and even drug treatment for those in need. During isolation, psychologists are also specially equipped with tools, such as decompression equipment, to help relieve bad moods. These psychological interventions also play an important role in the reduction of adverse psychological conditions. It has been shown that psychological intervention has a positive effect on maintaining the mental health of medical staff, and further research will also compare the specific effects of psychological intervention on mental state. Finally, this study will help to further improve psychological intervention systems so that they can better respond to public health crises and other emergencies.

### Restoration of the Human Environment

From the beginning of the pandemic, the Chinese government released COVID-19-related information using transparent methods. Understanding of the national COVID-19-related data is clear in media coverage. With the significant reduction in cases and deaths in the later period of the pandemic, the improved the humanity and custom environment gradually reduced the public panic. At the same time, the establishment of support by other family members enabled medical staff to have appropriate relaxation time and psychological acceptance of the regularization of COVID-19 prevention and control. In other countries, the importance of providing transparent and understandable information also has been discussed (Bäuerle et al., 2020). Therefore, from the perspective of psychological well-being, for future emergencies, China's mature treatment and information release methods are in line with individual psychological needs, which is also related to the reduction and mitigation of the poor mental states of medical staff.

### Shortcomings of the Study

This study had some limitations. First, the sample size of medical staff was sufficient for psychological state analysis and represented the status of medical staff in Liaoning Province. However, the sample source is unitary and there is no precise calculation of the sample size, which can lead to bias and less scientific data support. Second, although the survey method is anonymous, it is still possible that subjective dodge behavior will affect the results and presentation of objective facts, which impacts the final results and conclusions. Third, although conventional factors were selected, the factors studied may be one-sided and other factors affecting the occurrence of depression and anxiety may be ignored such as resilience, perceived organizational support, or social support satisfaction, studied by other studies (Cyr et al., 2021). Fourth, although the psychiatric medical staff have been investigated, no relevant investigations were conducted for medical staff in some special professions like other studies, such as health care professionals treating opioid use disorder (Blevins et al., 2021). Finally, this study found that the medical staff from ophthalmology and otolaryngology departments had the highest incidence of anxiety and depression, who are not from the departments involving in the examination and treatment of COVID-19 patients directly. However, because of the lack of basic research data before the COVID-19 outbreak, there is no way to make a comparison and explain whether the changes in the psychological status of the medical staff are related to the epidemic. Therefore, in the future, a larger sample of data from surveys is needed and more basic data is needed to get. Additionally, there is a need to minimize the generation of bias through more rigorous scientific design of research methods, improved processes and tools, and strengthened scientific analysis of more relevant factors.

Moreover, further analysis should be conducted through follow-up research, such as the effects of psychological interventions on mental status or the longitudinal impact of changes and differences in profession. In summary, continuous improvement of relevant research is needed to provide further scientific basis for policy making.

## Conclusion

The COVID-19 pandemic has hugely burdened health care, and mental symptoms need to be considered an important aspect. In the later phase of the pandemic in China, the prevalence of anxiety and depression of medical staff remains high. In addition to age, general factors such as gender, work position, profession, educational level, and other factors were found to be not related. On the contrary, concern for the psychological needs of medical staff is necessary through psychological counseling. Although there were some limitations in this study, the relevant data from this study can serve as a reference for further research, more comprehensive assessment of risk factors. Moreover, the sample survey suggests that medical staff, as a special group, show different psychological changes at various times during a stressful event. Thus, different psychologically oriented policy implementation that reduces adverse stress events is needed. It is also important to draw on past research experience in order to deal with current medical and health public events more quickly and efficiently.

## Data Availability Statement

The original contributions presented in the study are included in the article/supplementary material, further inquiries can be directed to the corresponding author.

## Ethics Statement

The studies involving human participants were reviewed and approved by the Ethics Committee of the People's Hospital of Liaoning Province. The patients/participants provided their written informed consent to participate in this study.

## Author Contributions

XY and RH: study design, data collection and analysis. JM: data collection and analysis. WF, LW, SS, HL, NW, XW, and ML: data collection. XY, JM, and RH: drafting of the manuscript. XY and RH: critical revision. All authors contributed to the article and approved the submitted version.

## Funding

The research fund was supported by Science and Technology Creative Zhiku Project of the Liaoning Science and Technology Association (LNKX2020B07).

## Conflict of Interest

The authors declare that the research was conducted in the absence of any commercial or financial relationships that could be construed as a potential conflict of interest.

## Publisher's Note

All claims expressed in this article are solely those of the authors and do not necessarily represent those of their affiliated organizations, or those of the publisher, the editors and the reviewers. Any product that may be evaluated in this article, or claim that may be made by its manufacturer, is not guaranteed or endorsed by the publisher.
